# Supplementary figures and images for: Analysis of (p)ppGpp metabolism and signaling using a dynamic luminescent reporter
Source: PLoS Genet. 2025 Aug 22;21(8):e1011691. doi: 10.1371/journal.pgen.1011691 (PMC12373219; doi:10.1371/journal.pgen.1011691)

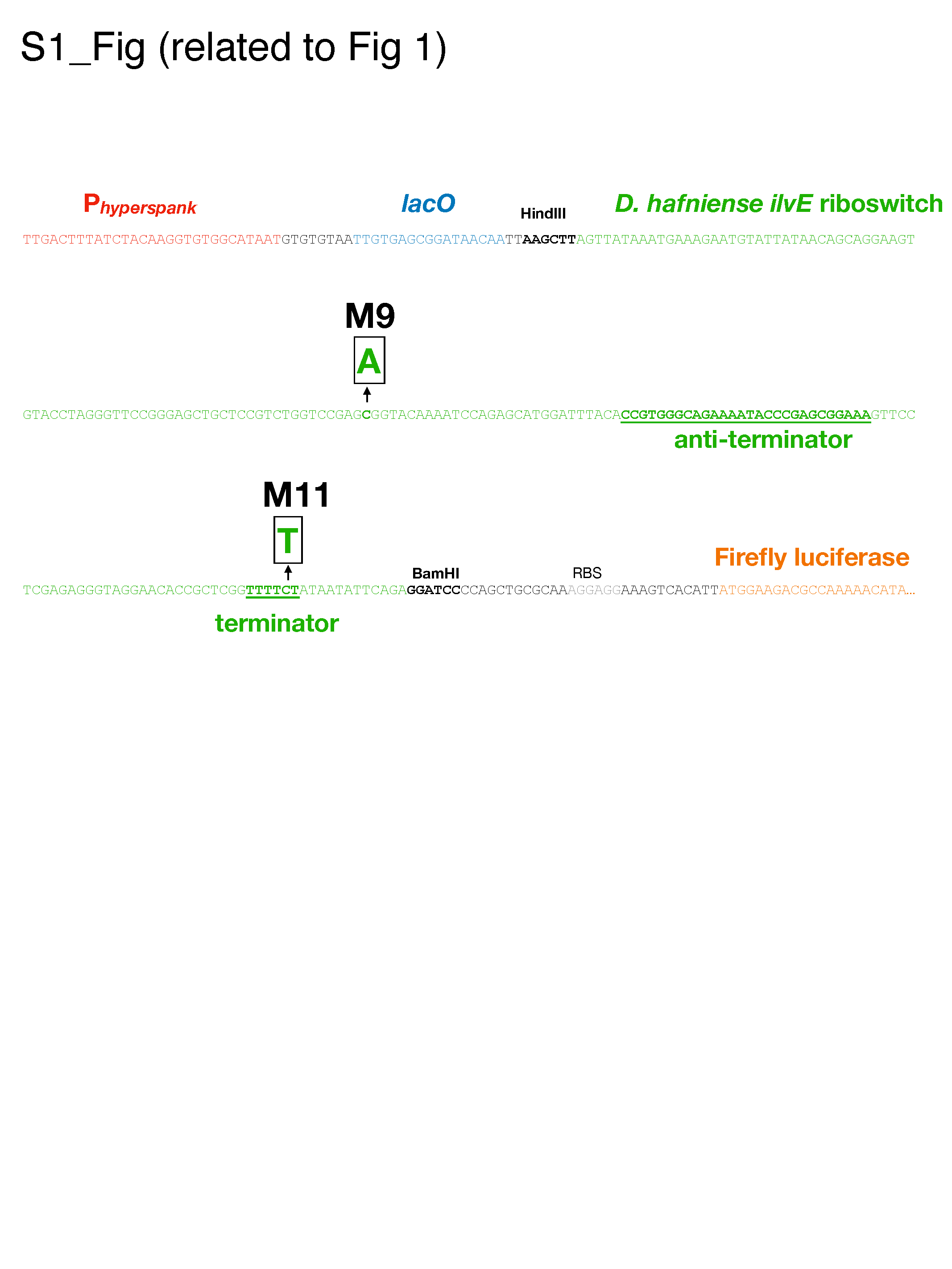

Supplement: S1 Fig — Shown is the Phyperspank promoter sequence (red), the lac operator binding sequence (blue) and the D. hafniense ilvE riboswitch gBlock sequence (green), with anti-terminator and terminator sequences bolded and underlined, and the M9 and M11 mutations from Sherlock et al 2018 annotated. In gray, the Shine-Delgarno ribosome binding sequence (RBS). In orange, the 5’ end of the firefly luciferase gene. Restriction enzymes HindIII and BamHI sites bolded. (TIFF) [file pgen.1011691.s001.tiff]

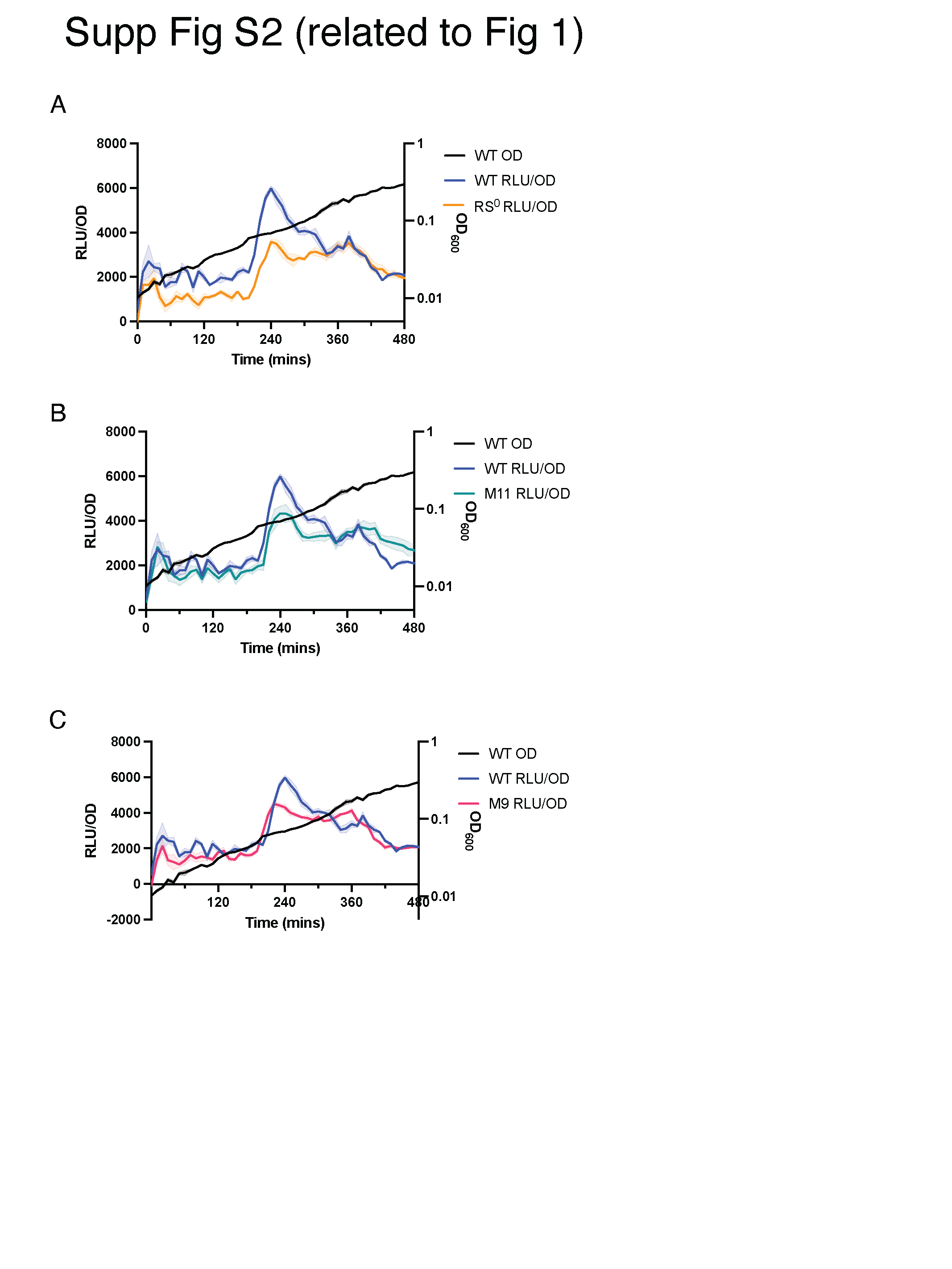

Supplement: S2 Fig — Luminescence (RLU/OD600) of: A, RsFluc (blue) and mutant RsFluc construct lacking an aptamer sequence (orange, RS0), B, RsFluc (blue) and M11 mutant RsFluc (green); and C, RsFluc (blue) and M9 mutant RsFluc (pink). Growth (OD600) (black). (TIFF) [file pgen.1011691.s002.tiff]

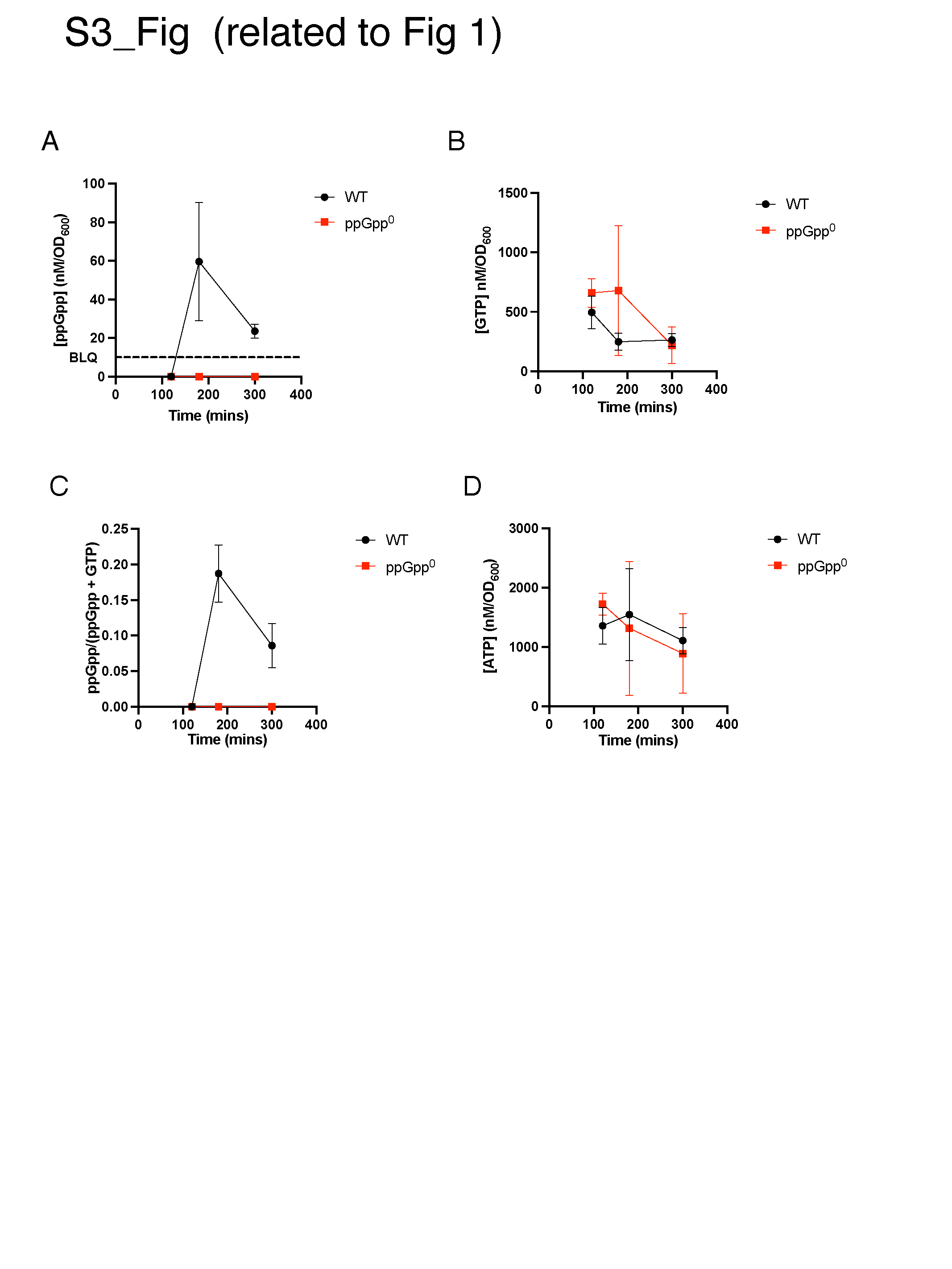

Supplement: S3 Fig — Nucleotide quantifications in the WT (black, JDB4496) and a (p)ppGpp0 background (red, JDB4512) of A) ppGpp, B) GTP, C) guanosine pools, and D) ATP, as analyzed via LC-MS collected at specified intervals. (TIFF) [file pgen.1011691.s003.tiff]

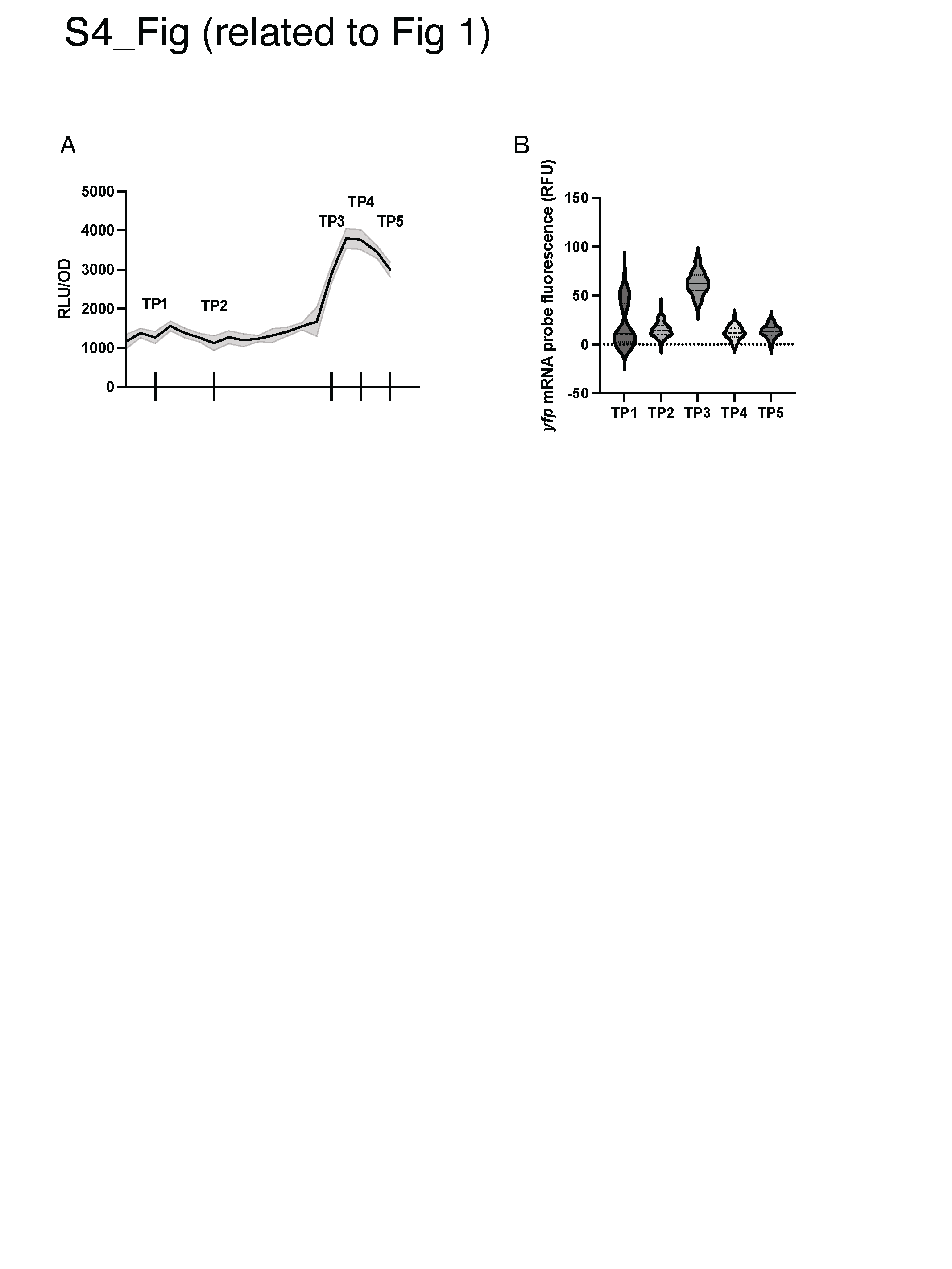

Supplement: S4 Fig — A) Samples of a strain carrying both RSFluc and ilvE-riboswitch-YFP reporters (JDB4623) were collected and analyzed via FISH at specified time points (TP1-TP5: TP1 = 80, TP2 = 120, TP3 = 200, TP4 = 220, TP5 = 240 mins) throughout the luminescence curve (RLU/OD) and imaged via fluorescent microscopy. B) The population distribution of single cell fluorescence at each time point (TP1, n = 202; TP2, n = 333; TP3, n = 481; TP4, n = 315; TP5, n = 197). (TIFF) [file pgen.1011691.s004.tiff]

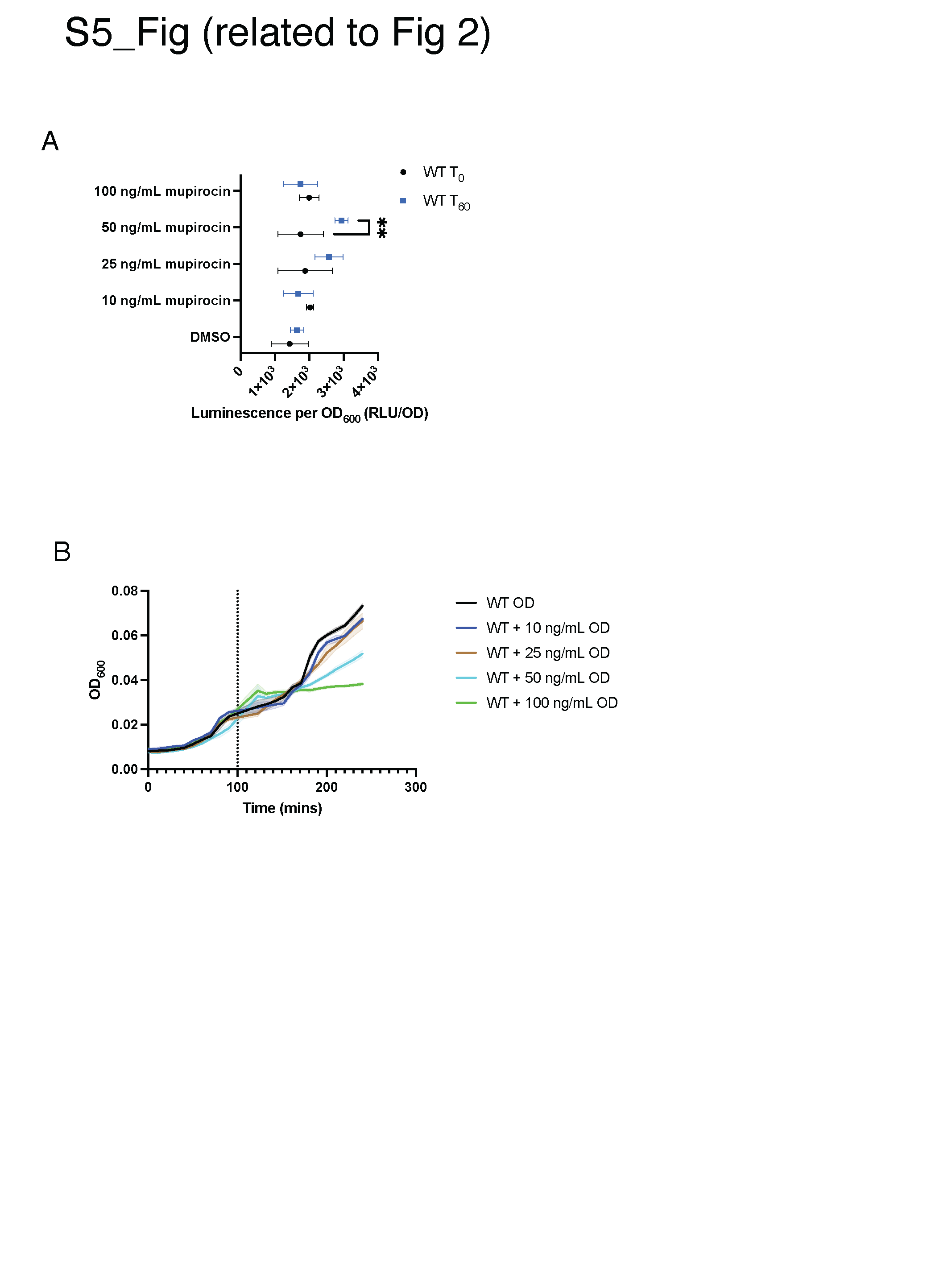

Supplement: S5 Fig — A, luminescence (RLU/OD600) of RsFluc (JDB4496) at start (T0) of treatment (black) and after 60 mins of treatment, T60 (blue) with varying concentrations of mupirocin. Significance determined by two-way ANOVA with multiple comparison, comparing T0 and T60 under each treatment. 50 ng/mL mupirocin (*) had a p-value of 0.0036 whereas other treatments were not significant. B, growth (OD600) of RsFluc before and after the time of mupirocin addition (dotted line) as treated with DMSO (black), 10 µg/mL (blue), 25 µg/mL (brown), 50 µg/mL (cyan), and 100 µg/mL (green) mupirocin. (TIFF) [file pgen.1011691.s005.tiff]

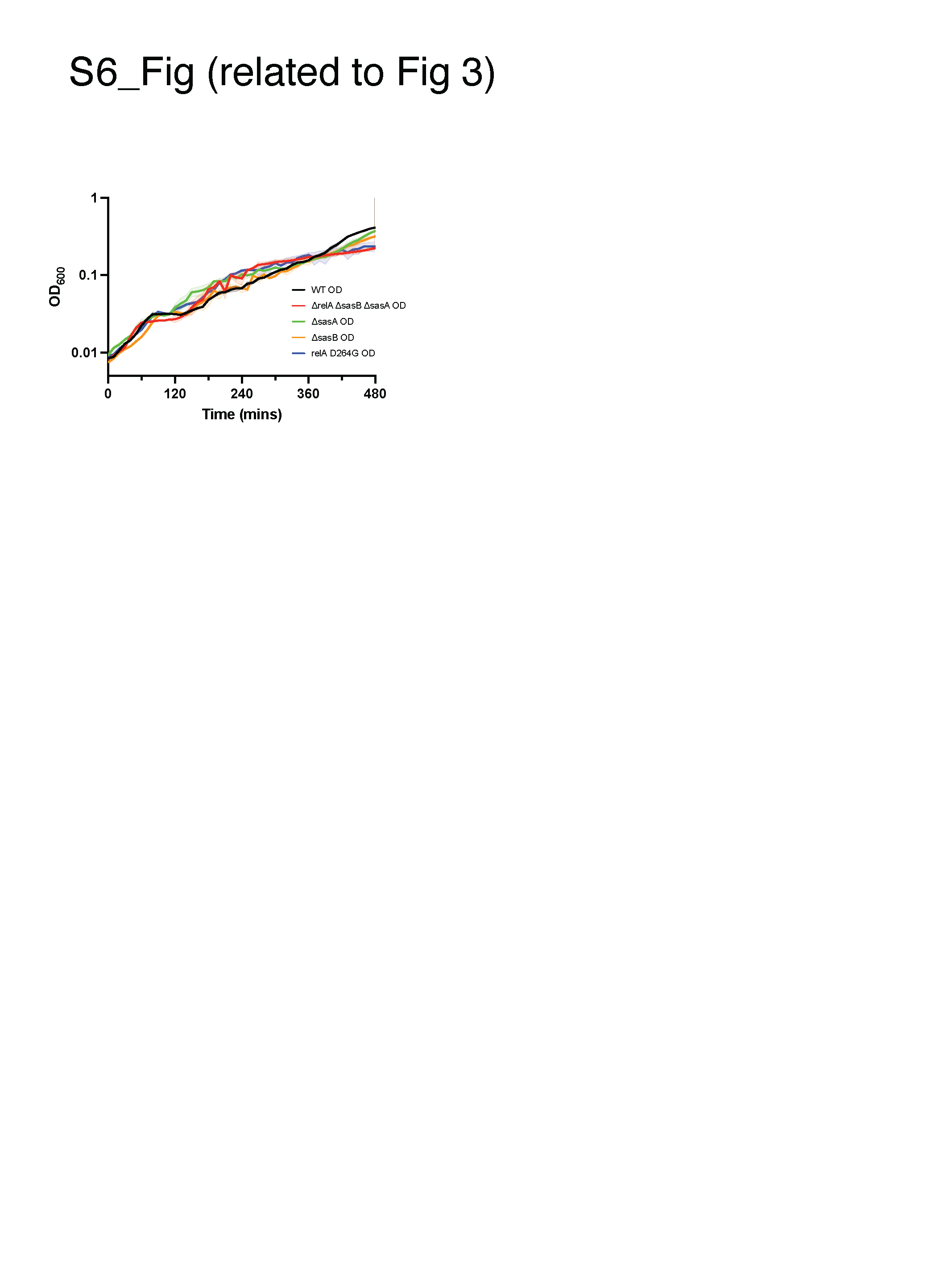

Supplement: S6 Fig — Growth (OD600) of wildtype (black, JDB4496), (p)ppGpp0 (red, JDB4512), ∆sasA (green, JDB4515), ∆sasB (orange, JDB4516), and relA-D264G (blue, JDB4741) strains. (TIFF) [file pgen.1011691.s006.tiff]

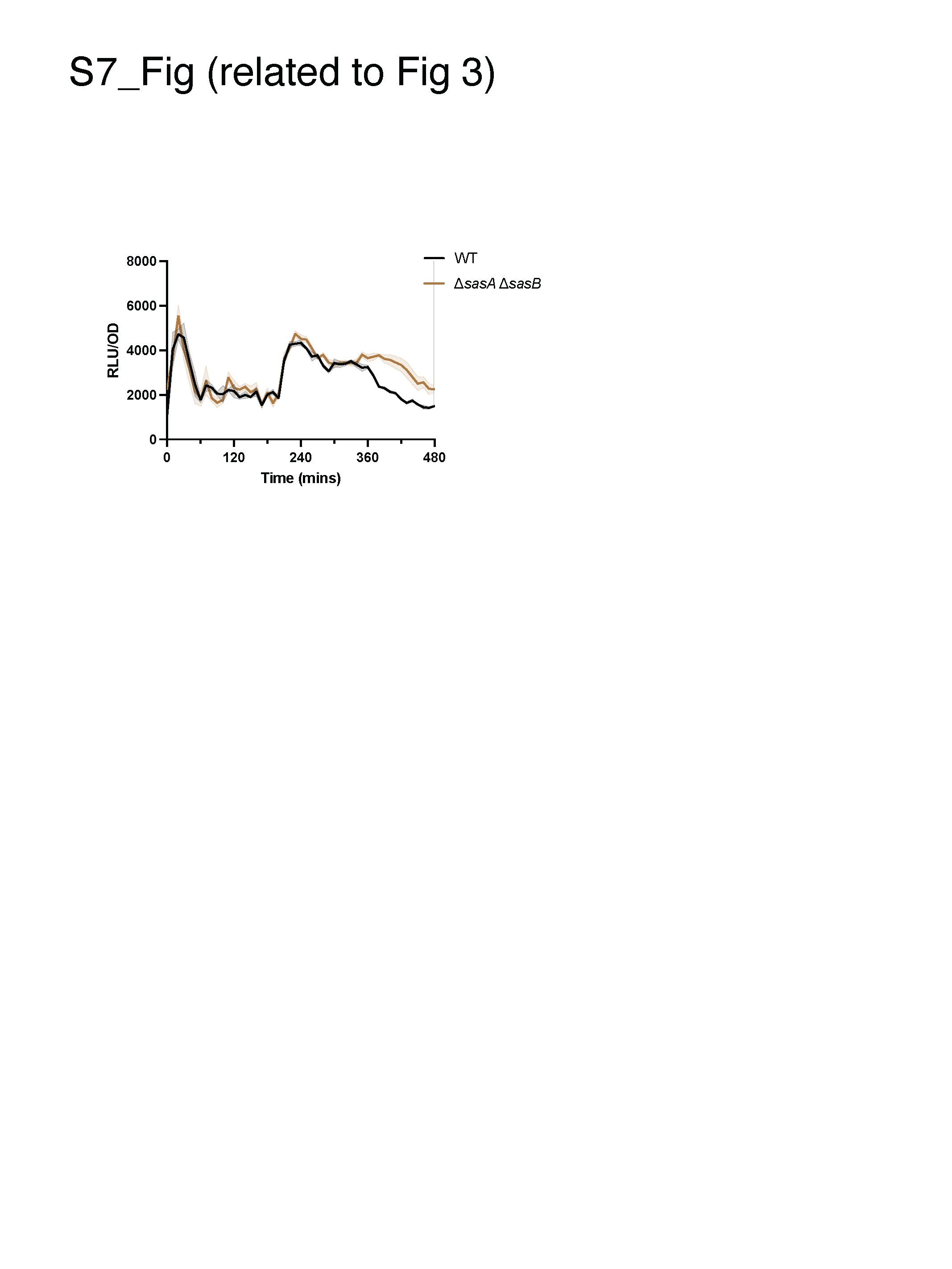

Supplement: S7 Fig — Luminescence (RLU/OD600) of RsFluc in WT (black, JDB4496) and ∆sasA∆sasB (gold, JDB4508) backgrounds. (TIFF) [file pgen.1011691.s007.tiff]

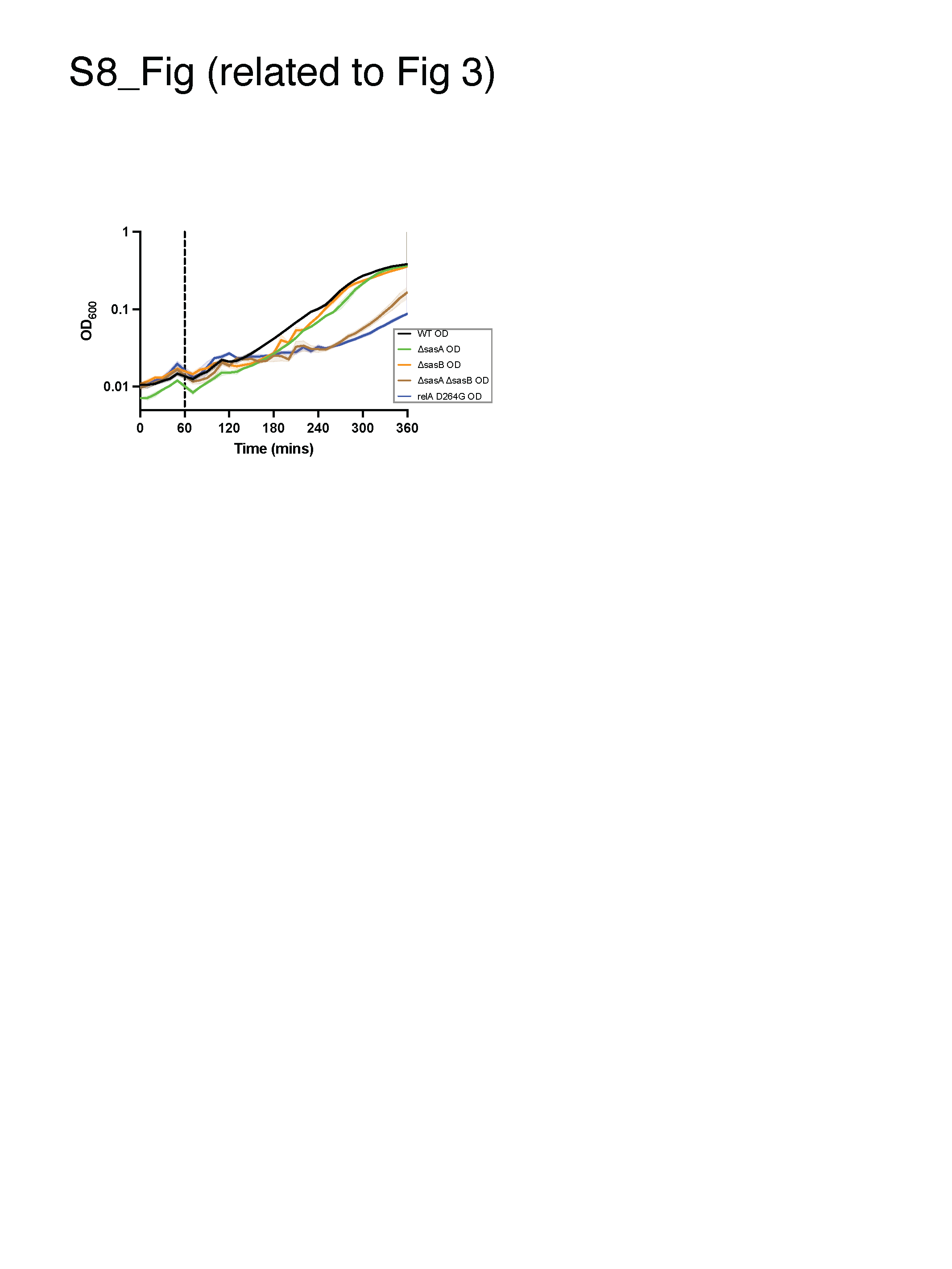

Supplement: S8 Fig — Luminescence (RLU/OD600) of RsFluc measured post nutrient downshift at T60 (dashed line) in WT (black, JDB4496), ∆sasA (green, JDB4515), ∆sasB (orange, JDB4516), ∆sasA∆sasB (gold, JDB4508), and relA-D264G (red, JDB4741) backgrounds. (TIFF) [file pgen.1011691.s008.tiff]

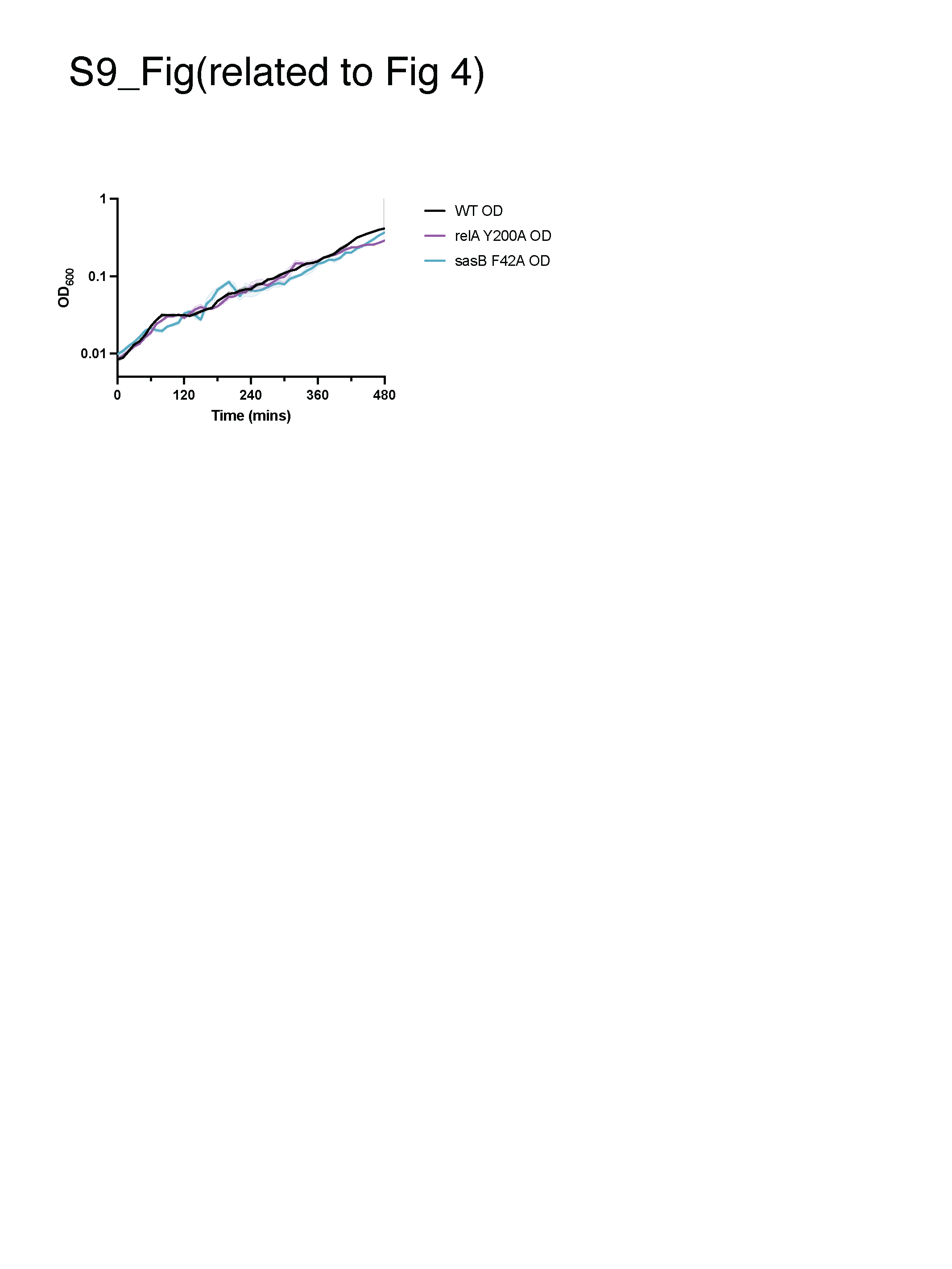

Supplement: S9 Fig — Growth (OD600) of wildtype (black, JDB4496), relA-Y200A (fuschia, JDB4528), and sasB-F42A (sky blue, JDB4711) strains. (TIFF) [file pgen.1011691.s009.tiff]

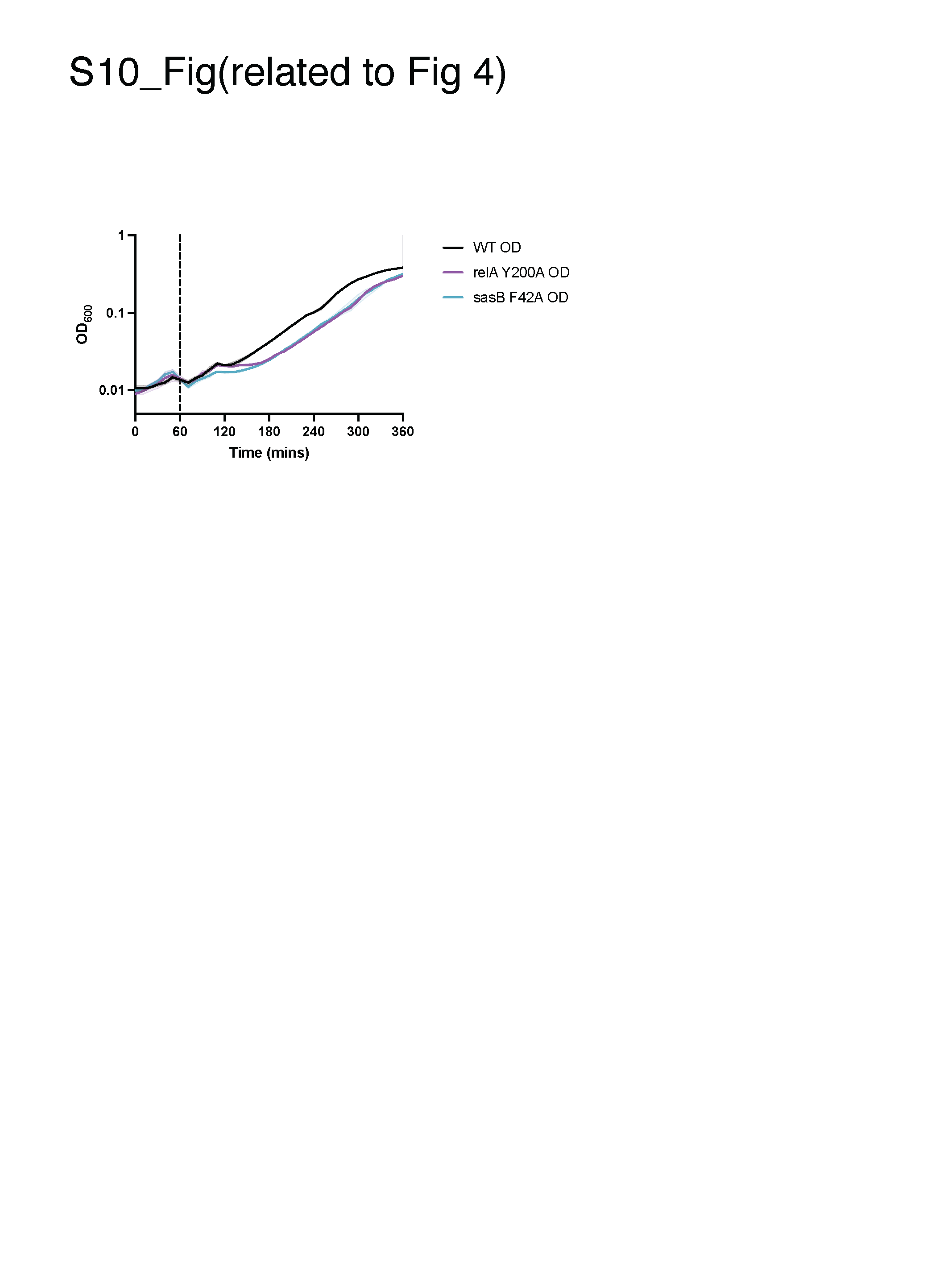

Supplement: S10 Fig — Growth (OD600) post nutrient downshift at T60, (dashed line) in wildtype (black, JDB4496), relA-Y200A (fuschia, JDB4528), and sasB-F42A (sky blue, JDB4711) strains. (TIFF) [file pgen.1011691.s010.tiff]

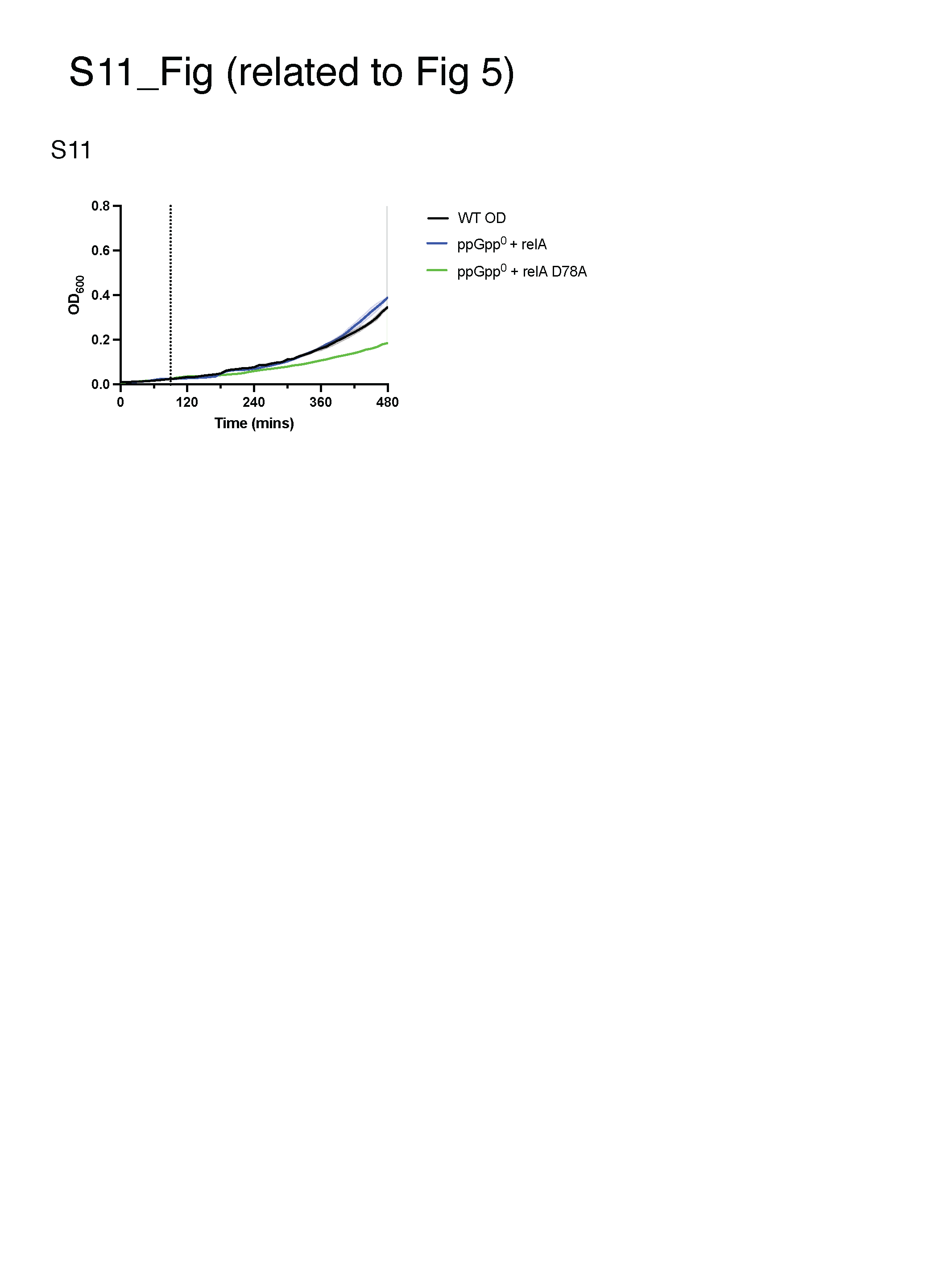

Supplement: S11 Fig — Growth (OD600) before and after 5 µg/mL bacitracin addition (dashed line) in WT (black, JDB4496), (p)ppGpp0 with inducible WT relA (blue, JDB4675), and (p)ppGpp0 with inducible relA-D78A (green, JDB4676) backgrounds. (TIFF) [file pgen.1011691.s011.tiff]

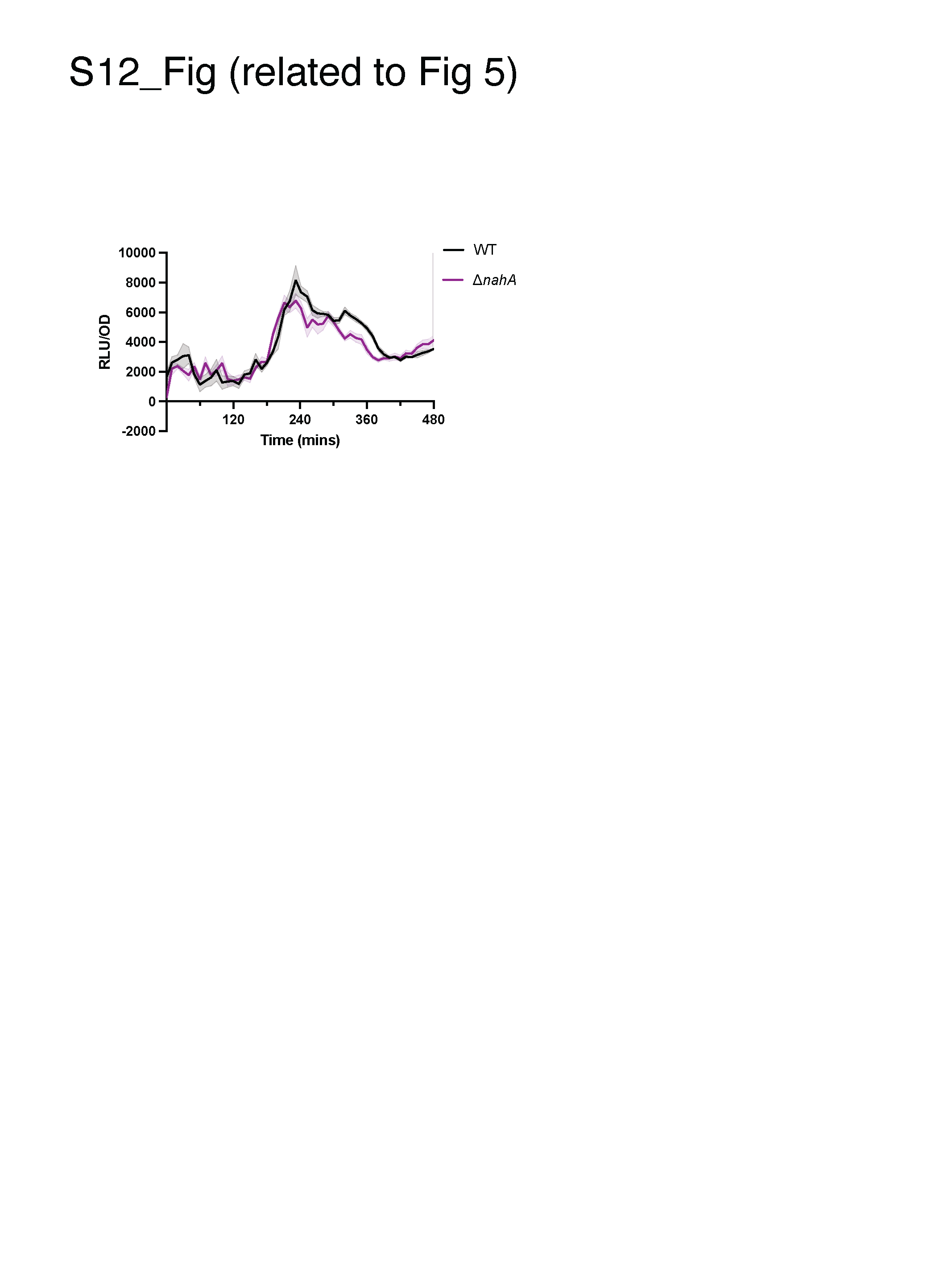

Supplement: S12 Fig — Luminescence (RLU/OD600) of RsFluc in wildtype (black, JDB4496) and ∆nahA (purple, JDB4567) backgrounds. (TIFF) [file pgen.1011691.s012.tiff]

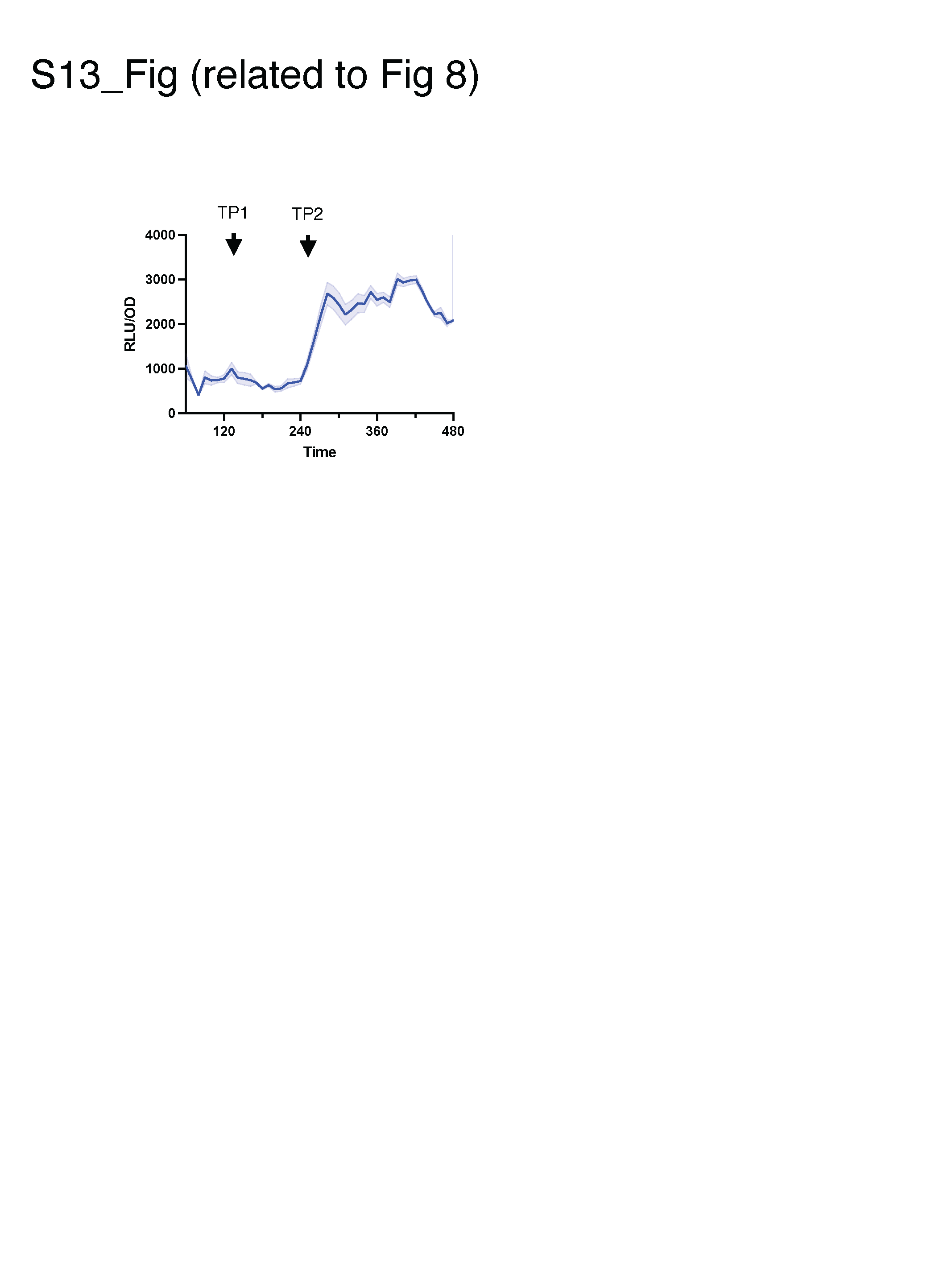

Supplement: S13 Fig — Luminescence (RLU/OD600) of RsFluc in wildtype (blue, JDB4496). Samples were taken at time points TP1 (120 min) and TP2 (240 min). (TIFF) [file pgen.1011691.s013.tiff]
