# Supplementary material for: Analysis of (p)ppGpp metabolism and signaling using a dynamic luminescent reporter
Source: PLoS Genet. 2025 Aug 22;21(8):e1011691. doi: 10.1371/journal.pgen.1011691 (PMC12373219; doi:10.1371/journal.pgen.1011691)
Supplement: S1 Table — (DOCX) [file pgen.1011691.s014.docx]

**S1_Table.**

| **Strain** | **Genotype** | **Source** |
| --- | --- | --- |
| JDB3 | *Prototroph (PY79)* | Lab collection |
| JDB1772 | *trpC2* | Lab collection |
| JDB4294 | *trpC2 relA::erm sasA(ywaC)::kan yjbM::tet* | (54) |
| JDB4300 | *trpC2 relA-Y308A* | (54) |
| JDB4440 | *trpC2 yjbM-F42A* | (54) |
| JDB4525 | *trpC2 relA-Y200A* | This study |
| JDB4729 | *trpC2 relA D264G* | This study |
| JDB4496 | *trpC2 sacA::P_hyperspank_-ilvE riboswitch-luciferase cmR* | This study |
| JDB4512 | *relA::erm ywaC::kan yjbM::tet sacA::P_hyperspank_-D. hafniense ilvE riboswitch-luciferase-cmR* | This study |
| JDB4631 | *trpC2 sacA::P_hyperspank_-M9+M11 riboswitch-luciferase-cmR* | This study |
| JDB4524 | *trpC2 sacA::Phyperspank-luciferase-cmR* | This study |
| JDB4599 | *trpC2 sacA::Phyperspank-M9 riboswitch-luciferase-cm* | This study |
| JDB4522 | *trpC2 sacA::Phyperspank-M11 riboswitch-firefly luciferase-cm* | This study |
| JDB4730 | *trpC2 sacA::Phyperspank-Clostridiales natA riboswitch-luciferase-cmR* | This study |
| JDB4731 | *trpC2 sacA::Phyperspank-Oxobacter pfennigii livK riboswitch-luciferase-cmR* | This study |
| JDB4508 | *trpC2 ywaC::kan yjbM::tet sacA::Phyperspank-D. hafniense ilvE riboswitch-luciferase-cmR* | This study |
| JDB4515 | *trpC2 ywaC::kan sacA::Phyperspank-D. hafniense ilvE riboswitch-firefly luciferase-cmR* | This study |
| JDB4516 | *trpC2 yjbM::tet sacA::Phyperspank-D. hafniense ilvE riboswitch-firefly luciferase-cmR* | This study |
| JDB4741 | *trpC2 relA D264G sacA::Phyperspank-D. hafniense ilvE riboswitch-luciferase-cmR* | This study |
| JDB4568 | *trpC2 ykuL::kan sacA::Phyperspank-D. hafniense ilvE riboswitch-luciferase-cmR* | This study |
| JDB4675 | *trpC2 relA::erm ywaC::kan yjbM::tet sacA::Phyperspank-D. hafniense ilvE riboswitch-luciferase-cmR amyE::PliaI-relA-specR* | This study |
| JDB4676 | *trpC2 relA::erm ywaC::kan yjbM::tet sacA::Phyperspank-D. hafniense ilvE riboswitch-luciferase-cmR amyE::PliaI-relA D78A-specR* | This study |
| JDB4567 | *trpC2 yvcI::erm sacA::Phyperspank-D. hafniense ilvE riboswitch-luciferase-cmR* | This study |
| JDB4711 | *trpC2 yjbM F42A sacA::Phyperspank-D. hafniense ilvE riboswitch-luciferase-cmR* | This study |
| JDB4528 | *trpC2 relA Y200A sacA::Phyperspank-D. hafniense ilvE riboswitch-luciferase-cmR* | This study |
| JDB4656 | *PY79 sacA::Phyperspank-D. hafniense ilvE riboswitch-luciferase-cmR* | This study |
| JDB4657 | *PY79 sacA::Phyperspank-M9+M11 riboswitch-luciferase-cmR* | This study |
| JDB4759 | *trpC2 sacA::PserA-luciferase-cmR* | This study |
| JDB4765 | *trpC2 sacA::PhomA-luciferase-cmR* | This study |
| JDB4792 | *trpC2 sacA::PilvB-luciferase-cmR* | This study |
| JDB4798 | *trpC2 sacA::PmetE-luciferase-cmR* | This study |
| JDB4793 | *trpC2 relA D264G sacA::PserA-luciferase-cmR* | This study |
| JDB4794 | *trpC2 relA D264G sacA::PhomA-luciferase-cmR* | This study |
| JDB4795 | *trpC2 relA D264G sacA::PilvB-luciferase-cmR* | This study |
| JDB4799 | *trpC2 relA D264G sacA::PmetE-luciferase-cmR* | This study |
| JDB4803 | *trpC2 sacA::PpurE-luciferase-cmR* | This study |
| JDB4804 | *trpC2 relA Y308A ywaC::kan yjbM::tet sacA::PpurE-firefly -cmR* | This study |
| JDB4303 | *trpC2 sacA::Phyperspank-D. hafniense ilvE. riboswitch-yfp-cmR* | This study |
| JDB4623 | *trpC2 sacA::Phyperspank-D. hafniense ilvE. riboswitch-yfp-cmR amyE::P Phyperspank-D. hafniense ilvE. riboswitch-luciferase-specR* | This study |
