# Supplementary material for: Analysis of (p)ppGpp metabolism and signaling using a dynamic luminescent reporter
Source: PLoS Genet. 2025 Aug 22;21(8):e1011691. doi: 10.1371/journal.pgen.1011691 (PMC12373219; doi:10.1371/journal.pgen.1011691)
Supplement: S2 Table — (DOCX) [file pgen.1011691.s015.docx]

**S2_Table S2.**

| **Plasmid** | **Description** | **Source** |
| --- | --- | --- |
| pSac-cm |  | Lab collection |
| pDR111 |  | Lab collection |
| pMINIMad2 |  | Lab collection |
| pGL3 |  | Lab collection |
| pEL47 | pSac-cm-Phyperspank | Lab collection |
| pMH9 | pSac-cm-Phyperspank-(p)ppGpp riboswitch-yfp | This study |
| pMH11 | pSac-cm-Phyperspank-(p)ppGpp riboswitch-firefly luciferase | This study |
| pMH13 | pSac-cm-Phyperspank-M9 riboswitch-firefly luciferase | This study |
| pMH15 | pSac-cm-Phyperspank-M11 riboswitch-firefly luciferase | This study |
| pMH16 | pSac-cm-Phyperspank-firefly luciferase | This study |
| pMH17 | pMINIMad2 relA Y200A | This study |
| pMH25 | pDG1730-Phyperspank-(p)ppGpp riboswitch-firefly luciferase | This study |
| pMH31 | pSac-cm-Phyperspank-M9+M11 riboswitch-firefly luciferase | This study |
| pMH39 | pDG1730-PliaI-relA | This study |
| pMH40 | pDG1730-PliaI-relA D78A | This study |
| pMH42 | pMINIMad2 relA D264G | This study |
| pMH45 | pSac-cm-Phyperspank-pppGpp riboswitch-firefly luciferase | This study |
| pMH46 | pSac-cm-Phyperspank-ppGpp riboswitch-firefly luciferase | This study |
| pMH50 | pSac-cm-PserA-firefly luciferase | This study |
| pMH67 | pSac-cm-PilvB-firefly luciferase | This study |
| pMH68 | pSac-cm-PmetE-firefly luciferase | This study |
| pSN27 | pSac-cm-PpurE-firefly luciferase | This study |
