# Supplementary material for: Analysis of (p)ppGpp metabolism and signaling using a dynamic luminescent reporter
Source: PLoS Genet. 2025 Aug 22;21(8):e1011691. doi: 10.1371/journal.pgen.1011691 (PMC12373219; doi:10.1371/journal.pgen.1011691)
Supplement: S3 Table — (DOCX) [file pgen.1011691.s016.docx]

**S3_Table**

**Oligos**

| Source | Number | Name | Sequence |
| --- | --- | --- | --- |
| (27) | MES153 | IDT G-block containing the WT D. hafniense ilvE riboswitch and controlled by the B. subtilis lysC promoter, for serving as a dsDNA template for in vitro transcription termination assays | GCGCACATGAGAATTCCAGCGACGCTGTTGATCCTTTTAAATAAGTCTGATAAAATGTGAACTAAAGTTATAAATGAAAGAATGTATTATAACAGCAGGAAGTGTACCTAGGGTTCCGGGAGCTGCTCCGTCTGGTCCGAGCGGTACAAAATCCAGAGCATGGATTTACACCGTGGGCAGAAAATACCCGAGCGGAAAGTTCCTCGAGAGGGTAGGAACACCGCTCGGTTTTCTATAATATTCAGAGGATCCCCAGCTGCGC |
| This study |  | ilvE riboswitch gBlock | AGTTATAAATGAAAGAATGTATTATAACAGCAGGAAGTGTACCTAGGGTTCCGGGAGCTGCTCCGTCTGGTCCGAGCGGTACAAAATCCAGAGCATGGATTTACACCGTGGGCAGAAAATACCCGAGCGGAAAGTTCCTCGAGAGGGTAGGAACACCGCTCGGTTTTCTATAATATTCAGAGGATCCCCAGCTGCGC |
| This study |  | natA riboswitch gBlock | ACTGTCAAGCTTCAATCATATTATAATATTAACAATAAGAGAGTGTATCTAGGGTTCCGGTCAATAGATGTCTGGTCCGAGCGATACAGGATTTCAATCTACACTTTTAGGAAAAAAGCCTAAAGGACGAGTCTCTGCAAAGAGATTTGTTCTTGGGCTTTATTTTTTTATCTTTATTTAAGGGGGCGGCCGCTGAGTC |
| This study |  | livK riboswitch gBlock | AGTCTGAAGCTTTAATTTATAATGACAAATTAATTATGTTGATAAGAGGCTAACTAGGGTTCCGATGGTTTCATGCTGGTCCAAGCGTTAGCAAACATACTGAGGAAGGTATGTTACACCGTTAGTACAAAAGGCTCGGCGGGAGGTCTTATCCTTAGATGGACAAGGCCCCCCTACGAGTTTTTTTATTAAACTTGTTGACATAATGTGTCATTATATGGCGGCCGCTGACTG |
| This study |  | ilvE riboswitch F1 F | CCCGGGAAGCTTAGTTATAAATGAAAGAATGTATTATAACAGCAG |
| This study |  | rbs-YFP F2 R | CCCGGGGCTAGCTTTATTTATACAGTTCGTCCATACCGTG |
| This study |  | ilvE riboswitch F1 R | GCTCATAATGTGACTTTCCTCCTTTGCGCAGCTGGGGATCCTC |
| This study |  | rbs-YFP F2 F | ATATTCAGAGGATCCCCAGCTGCGCAAAGGAGGAAAGTCACATTATGAGCA |
| This study | MH53 | pSacA with luciferase F | GCGGAAAGATCGCCGTGTAAAGCTAGCCGCATGC |
| This study | MH54 | pSacA with luciferase R | ATGTTTTTGGCGTCTTCCATAATGTGACTTTCCTC |
| This study | MH55 | luciferase in pSacA F | CAAAGGAGGAAAGTCACATTATGGAAGACGCCAAAA |
| This study | MH56 | luciferase in pSacA R | TAGCTTGCATGCGGCTAGCTTTACACGGCGATCTTTC |
| This study | MH145 | M9 Q5 SDM F | GGTCCGAGAGGTACAAAATCCAGAGC |
| This study | MH146 | M9 Q5 SDM R | AGACGGAGCAGCTCCCG |
| This study | MH50 | M11 Q5 SDM F | CCGCTCGGTTTTTTATAATATTCAG |
| This study | MH51 | M11 Q5 SDM R | CTGAATATTATAAAAAACCGAGCGG |
| This study | MH65 | luciferase in pSac-cm-Physpank F | ATTAAGCTTaggaggAGCCACCATG |
| This study | MH66 | luciferase in pSac-cm-Physpank R | CGGCTAGCTTACACGGCGATCTTT |
| This study | MH22 | relA EcoRI F pMINIMAD2 | gggcccgaattcCATCTTTCGTTTTTTTCTTG |
| This study | MH23 | relA BamHI R pMINIMAD2 | gggcccggatccTGGGCTTCATTCGTTTTG |
| This study | MH67 | relA Y200A F | ACCCTCAGCAAgcgTACAGAATTGT |
| This study | MH68 | relA Y200A R | ACAATTCTGTAcgcTTGCTGAGGGT |
| This study | MH167 | EcoRI PliaI F | CAGTCAGAATTCATTGGCCAAAGCAGAAAGGTCC |
| This study | MH168 | SalI PliaI R | CTGACTGTCGACTCGTTTTCCTTGTCTTCATCTTATAC |
| This study | MH57 | relA D78A F | ATTTTTGCACgcgGTCGTGGAAG |
| This study | MH58 | relA D78A R | CTTCCACGACcgcGTGCAAAAAT |
| This study | MH121 | Pspac RS luc EcoRI F | GTCAAACATGAGAATTCGACTCTCTAGCTTG |
| This study | MH122 | Pspac RS luc EcoRI R | gggaaagaattcCTCACATTAATTGCGTTGCGCTCAC |
| This study | MH205 | natA riboswitch gBlock F | ACTGTCAAGCTTCAATCATATTATAATATTAACAATAAG |
| This study | MH206 | natA riboswitch gBlock R | GACTCAGCGGCCGCC |
| This study | MH207 | livK riboswitch gBlock F | AGTCTGAAGCTTTAATTTATAATGACAAATTAATTATGTTG |
| This study | MH208 | livK riboswitch gBlock R | CAGTCAGCGGCCGCCA |
| This study | MH209 | NotI luciferase F | CTGACAGCGGCCGCAAAGGAGGAAAGTCACATTATG |
| This study | MH210 | NheI luciferase R | GCATGCGGCTAGCTTTACAC |
| This study | MH231 | BamHI PserA F | CTGACAGGATCCCGAATCCTGCTGTAAAGACAGC |
| This study | MH232 | HindIII PserA R | TGACTGAAGCTTGACAAACTAAATATCTGATAATTTAACATATTCTC |
| This study | MH265 | BamHI PilvB F | CTGACAGGATCCGAAATTGAAATGGATTG |
| This study | MH266 | NheI PilvB R | CTGACAGCTAGCGGATTTTCATCCTTTAAAGATCATC |
| This study | MH271 | BamHI PmetE F | CAGTCAGGATCCCACATCATGTAAATAAAAATTTCAAATTC |
| This study | MH272 | HindIII PmetE R | AGTCTGAAGCTTTTATATGTAAAACACTCTCTTTCACC |
| This study | NSN139 | PpurE luc F | ccggcgctcaggatccGGAAATTGATCTAAAACACGAAC |
| This study | NSN140 | PpurE luc R | ACCGGAATGCCAAGCTTAATGCTTTTGTTTTCAGAAAAT |
| This study |  | *yfp* FISH probe 1 | aaagttcttctcctttacgc |
| This study |  | *yfp* FISH Probe 2 | agaattgggacaactccagt |
| This study |  | *yfp* FISH Probe 3 | cccattaacatcaccatcta |
| This study |  | *yfp* FISH Probe 4 | cctctccactgacagaaaat |
| This study |  | *yfp* FISH Probe 5 | gtaagttttccgtatgttgc |
| This study |  | *yfp* FISH Probe 6 | gtagttttccagtagtgcaa |
| This study |  | *yfp* FISH Probe 7 | acaagtgttggccatggaac |
| This study |  | *yfp* FISH Probe 8 | tgaacaccccaagtcaaagt |
| This study |  | *yfp* FISH Probe 9 | tcatgccgtttcatatgatc |
| This study |  | *yfp* FISH Probe 10 | gggcatggcactcttgaaaa |
| This study |  | *yfp* FISH Probe 11 | ttctttcctgtacataacct |
| This study |  | *yfp* FISH Probe 12 | gttcccgtcatctttgaaaa |
| This study |  | *yfp* FISH Probe 13 | tgacttcagcacgtgtcttg |
| This study |  | *yfp* FISH Probe 14 | taacaagggtatcaccttca |
| This study |  | *yfp* FISH Probe 15 | ataccttttaactcgattct |
| This study |  | *yfp* FISH Probe 16 | gaatgtttccatcttcttta |
| This study |  | *yfp* FISH Probe 17 | ttgtattccaatttgtgtcc |
| This study |  | *yfp* FISH Probe 18 | gtttatctgcagtgatgtat |
| This study |  | *yfp* FISH Probe 19 | tgagctttgattccattctt |
| This study |  | *yfp* FISH Probe 20 | ccatcttcaatgttgtgtct |
| This study |  | *yfp* FISH Probe 21 | atggtctgctagttgaacgc |
| This study |  | *yfp* FISH Probe 22 | cgccaattggagtattttgt |
| This study |  | *yfp* FISH Probe 23 | ttgtctggtaaaagggcagg |
| This study |  | *yfp* FISH Probe 24 | cagattgtgtggacaggtaa |
| This study |  | *yfp* FISH Probe 25 | ttttcgttgggatctttcga |
| This study |  | *yfp* FISH Probe 26 | aagaaggaccatgtggtctc |
| This study |  | *yfp* FISH Probe 27 | tcccagcagctgttacaaac |
| This study |  | *yfp* FISH Probe 28 | ttatacagttcgtccatacc |
