# Supplementary material for: Analysis of (p)ppGpp metabolism and signaling using a dynamic luminescent reporter
Source: PLoS Genet. 2025 Aug 22;21(8):e1011691. doi: 10.1371/journal.pgen.1011691 (PMC12373219; doi:10.1371/journal.pgen.1011691)
Supplement: Attachment — Submitted filename: Rebuttal.pdf [file pgen.1011691.s019.pdf]

Comments to the Authors:
Please note here if the review is uploaded as an attachment.

Reviewer #1: In this manuscript, Hydron et al. designed and synthesized a riboswitch-based luciferase reporter for (p)ppGpp, the alarmone that senses amino acid limitation, in Bacillus subtilis. Using a multipronged approach, they first confirmed that the luminescence intensity indeed reflects the (p)ppGpp level in cells. Subsequent, they used this reporter to test various aspects of known (p)ppGpp regulation and the results largely agreed with the literature reports except the absence of decrease in GTP pool when (p)ppGpp increases. Overall, I found the reporter very useful – as the authors mentioned in the discussion, riboswitch reporters have now been increasingly used to measure intracellular concentration of second messengers, and there is a need for more reporters for (p)ppGpp in particular. The potential application in mouse model or in natural bacterial communities is also attractive. The manuscript is thorough; the logic is clear; and the assays well designed and executed. I support the publication of the manuscript in PloS Genetics. There are following comments I have on the technical aspect that the authors may wish to consider in a revised manuscript.

Major Comments:

1. Interpretation of (p)ppGpp synthase deletion effects: The authors use a (p)ppGpp^0 strain (ΔrelAΔsasAΔsasB) to show that RsFluc luminescence is strongly reduced (Line 114-117, Figure 1B). While this supports the idea that RsFluc primarily reports (p)ppGpp abundance, it may be worth acknowledging that deletion of relA, sasA, and sasB also broadly alters cell physiology, and global changes in transcription or translation could secondarily affect luciferase expression.

Thank you for that comment. In fact, we developed the RsFluc^{mut} reporter as a control for these secondary physiological effects. We have altered the text to include this consideration in both the results (lines 123-126) and discussion sections (lines 295-307).

2. Timing of luciferase reporter responses: In Figure 2D, RsFluc luminescence (directly sensing (p)ppGpp) and PurE-luc luminescence (reflecting transcriptional activation) are compared. Since both RsFluc and PurE-luc are luciferase-based reporters, I appreciate that their kinetics can be very rapid. However, because PurE-luc reflects transcriptional activation downstream of (p)ppGpp signaling, whereas RsFluc reflects direct sensing of (p)ppGpp abundance, I would still expect a short but noticeable delay in PurE-luc luminescence relative to RsFluc luminescence. In the current figure, the two signals change almost simultaneously (also mentioned in Line 178). Could the authors comment on the expected timing resolution and whether the sampling interval allows detection of a transcriptional delay? Explicitly commenting on this point would help readers better understand the kinetics of (p)ppGpp signaling versus downstream gene expression.

We were careful not to overinterpret the kinetics of RsFluc compared to P_{purE}-luciferase because there are more factors contributing to the *purE* operon expression than (p)ppGpp abundance. For example, the PurR repressor is derepressed by PRPP binding. In Fig. 2D, there is a (p)ppGpp independent decrease in luminescence seen in both the WT and (p)ppGpp^0 reporter, likely a result in a synchronized fall in PRPP levels in both strains. The next decrease occurs around the time that RsFluc begins increasing, suggesting that these cultures are experiencing increase (p)ppGpp synthesis. We also saw that (p)ppGpp dependent amino acid biosynthetic operon expression

increased at the same time as RsFluc (Fig. 7), suggesting that (p)ppGpp signaling can enact rapid change in cellular gene expression. However, we cannot argue with certainty that chances in (p)ppGpp abundance precedes the change in gene expression using these population-level measures like luciferase. One way to investigate this more conclusively is timelapse microscopy using one fluorophore to assay (p)ppGpp signaling and another for a response gene, but that is beyond the scope of the present study.

The sampling interval is 10 minutes for these experiments which is around two half-lives of the luciferase enzyme. This interval could be shortened to allow for increased temporal resolution. However, we found that shortening increased reporter noise.

3. OPP labeling interpretation (Figure 8): The OPP labeling experiments nicely demonstrate a decrease in active translation upon (p)ppGpp accumulation. However, I wonder whether additional controls could strengthen the interpretation. In particular:

● Since OPP labeling efficiency and uptake could vary depending on membrane status or cell stress, did the authors assess cell viability/growth or membrane integrity during the OPP labeling period?

We did not directly assess viability or membrane integrity during the OPP labeling period. The OPP incorporation step is performed at exponential and early transition time points where the bacteria are exhibiting robust growth and presumably not highly stressed.

● Given that OPP only measures global nascent protein synthesis, is it possible that selective translation of stress-induced proteins persists even when global rates drop?

We agree that this is a very real possibility. In fact, we have in fact observed such a phenomenon, as reported in a manuscript presently under review.

● Did the authors check whether OPP labeling efficiency is comparable across conditions (wild-type vs. mutant, stressed vs. unstressed)?

We did not test for this (also see discussion above). After incorporating OPP, cells get fixed and membrane permeabilized using mild detergent and we assumed labeling efficiency is homogeneous across different conditions. We do assume that OPP uptake is similar in wt and mutant strains, although it is difficult to be certain of this as the mechanism of puromycin uptake (to our knowledge) is not understood. An alternative labeling method based on homopropargylglycine (HPG) exists. However, HPG is a methionine analog and so is likely to be taken up through amino acid import machinery whose expression is affected by (p)ppGpp abundance in *E. coli*. (PMID: 370095, 18039766)

● It would improve the clarity by briefly explaining why the authors picked 120 min and 240 min as TP1 and TP2, respectively.

We picked these time points as they reflected times during which growth was rapid and reporter signal was low (TPI) and times where growth had fallen and reporter signal was high (TPII). We used these time points to argue that the (p)ppGpp response may regulate changes in growth by slowing global protein synthesis rates, as was shown in a previous article from our lab. We clarify this in the revised text, line 249-51.

Minor Comments:
1. Figure 1A: The authors should provide more details about the RsFluc construct either in the figure or the figure legend (e.g., Is the red section in the ppGpp riboswitch a RBS?). Also, there is an unintended break or gap in the line within the riboswitch region and a short red line is present immediately after the riboswitch, which does not seem to correspond to any described feature. Suggest correcting these minor artifacts to improve figure clarity.

We have added a new supplementary figure containing the sequence of the reporter upstream of the luciferase gene, annotated with sequence features. See Fig. **S1.**

2. Line 191: Italicizing sasA and sasB in "… or the combination ΔsasAΔsasB …".

Corrected, see line 196.

3. Line 205-207: "…the magnitude of the increase was not substantially affected, indicating that Rel Y200A likely does not impair synthase activity.". This statement seems only true during a nutrient downshift but not during normal growth. It would increase the clarity by specifying the condition.

Corrected, see line 212.

4. Line 283: "… as the mupirocin used in the earlier studies rapidly and strongly simulates (p)ppGpp abundance in <5 min." There is a small typo where "simulates" should be corrected to "stimulates".

Corrected, see line 289.

5. It would improve the clarity of the bar graphs if individual data points (e.g., scatter overlay) were shown, allowing readers to assess the variability among replicates.

Figure 2B has been corrected to show individual data points. Figures 8C and 8D have been changed to violin plots to better illustrate variability.

6. While RsFluc activity closely tracks expected (p)ppGpp dynamics, it is worth noting that (p)ppGpp broadly affects transcription, translation, and metabolic remodeling. I suggest briefly

discussing the possibility of these indirect effects on reporter expression, acknowledging that while RsFluc predominantly reflects (p)ppGpp levels, secondary physiological changes could modulate reporter expression to a small extent.

We have added a brief discussion about this. Please see lines 295-307.

Reviewer #2: This study by Hydorn et. al. develops a riboswitch-based luminescent reporter for the alarmone ppGpp that enables temporal monitoring of levels in live B. subtilis cells. Importantly, besides showing that the reporter works through use of mutants (ppGpp-0 strain, synthase knockouts, rel mutants that affect synthase or hydrolase activity), the researchers were able to observe ppGpp changes in response to amino acid downshift and diauxic shift; use riboswitch variants to separately measure pppGpp and ppGpp levels; correlate the riboswitch reporter to promoters known to be activated by ppGpp. The riboswich-based reporter can be an important tool for other researchers in the field who have sought to study ppGpp dynamics in B. subtilis; however I have the following questions about the study:

1. The researchers do not provide the sequence of their promoter-riboswitch-luciferase reporter construct, they only show a diagram of the construct in Fig. 1A. This will make it difficult for other researchers to make use of this tool.

A supplementary figure has been added to include the sequence of the reporter with features annotated. **See Fig. S**1

2. The sequence of the double riboswitch mutant is not provided, and data on this mutant appear to show that it reduces the signal but does not fully disrupt the riboswitch response. Is there a more "null" riboswitch mutant that could be used?

A supplementary figure has been added to include the sequence of the double riboswitch mutant. **See Fig. S1.** In Fig. 4B of Sherlock et al., 2018, they observed readthrough transcription of the mutant reporters, though the levels did not change with increasing amounts of (p)ppGpp. We therefore expected some level of read-through transcription of this double mutant riboswitch. Given the absence of a structure of the *ilvE* riboswitch bound to (p)ppGpp, we are not able identify mutations that would potentially be even more “null”. However, as we now note (line 299-301), attenuation of the RsFluc^{mut} reporter is similar to that observed with mutant RsFluc reporter lacking the entire RS element (RS^0, Fig. S1A), suggesting that RsFluc^{mut} is pretty “null”.

3. Given the strong effect of ppGpp on translation, which the researchers also demonstrate in Figs 8 and S11, they should address whether the drop observed in RLU/OD signal after the peak that typically is at ~240 min may be due to ppGpp decreasing reporter protein synthesis, rather than an actual drop in ppGpp levels. How is the reporter not going to be affected by reduced protein synthesis if its half-life is ~5 mins?

We agree that the reporter will likely be affected by reduced protein synthesis, but this would dampen the signal rather than artificially inflate the signal we are seeing at our time point (as is now

discussed lines 303-307). But, to demonstrate that changes in luminescence reflected actual changes in reporter transcription rather than changes in protein synthesis levels, we employed FISH microscopy with fluorescent probes that hybridized to *yfp* mRNA under the Phyperspank-(p)ppGpp RS regulation in a strain that also contained the RS-Fluc reporter. We monitored luminescence via the plate reader and collected cells before, during, and shortly after the major spike in luminescence, hybridized with the probes, and imaged. We saw that there was low hybridization before and after the spike, and high hybridization during the spike, indicating that the luminescence is reflecting *luc* mRNA levels, and thereby actual (p)ppGpp levels. See **fig S4**.

4. Some additional comments: (i) the authors should discuss the ATP and oxygen dependence of the firefly luciferase reporter, which may complicate imaging

The oxidation of luciferin to oxyluciferin by luciferase is a reaction that depends on ATP and oxygen. We did collect samples for HPLC-MS analysis of nucleotide levels throughout growth and differing luminescence levels and saw that ATP levels were consistent across different time points (Fig. S2D). While we didn't directly assay for O_2 throughout growth, the conditions were kept constant with shaking to encourage culture aeration during luminescent growth experiments. Consistent with the cultures being adequately aerated, the WT P_{purE}-*luciferase* reporter exhibited peak luminescence (Fig. 2D) in stationary phase of growth, when cultures are at their densest.

(ii) "the first time" claim in summary - please see 10.1002/anie.202111170;

Thank you for pointing this out. Corrected, see line 40.

(iii) "adds them to the 3' carbon" - should be 3' hydroxyl

Corrected, see line 56

Reviewer #3: ppGpp is a profoundly important signaling molecule used by diverse bacterial species. In this study, the authors built a biosensor using a ppGpp-sensing riboswitch, thereby allowing them to investigate the relative intracellular dynamics of ppGpp. They validated this biosensor through a collection of mutant strains and growth conditions. Additionally, the authors demonstrated that the ppGpp-sensing reporter fusion could be used to gain insight into the functional role(s) and significance of different synthase and hydrolase enzymes. The manuscript is clear and the data overall supports the authors' claims. I am confident this manuscript will be well-cited and that other researchers will quickly adopt usage of the ppGpp riboswitch reporter fusion. I have only a few minor comments.

Line 72: remove PMID number.

Corrected

Lines 473-4: An additional sentence including brief description of culture medium (or a reference) would improve this methods subsection.

Please see lines 493-5.

Fig 2B: Is it necessary to include the key if the labels will also be included on the x-axis? Also, since “RsFluc-mut” and “M9+M11” refer to the same reporter, choosing one nomenclature will make it easier to understand for the reader. Similarly, a different nomenclature style is used in panel 2D (“ppGpp RS-luciferase”), so coordinating the labels across the figures would help the reader.

Corrected. See changes to Fig. 2B and 2D.

Fig 2C: The spike in ppGpp signal as the bacterium switches carbon sources during diauxic growth in interesting; however, since the flattening of the growth curve is essentially a temporary stationary phase, wouldn’t this result imply that a spike in ppGpp would coincide with depletion of the glucose even when a second carbon source had not been included?

Yes, we agree. Consistently, in the culture grown in 2.5 mg/mL glucose (Fig. 2C, dark black), there is a slight increase in reporter signal as it approaches stationary phase.

Fig 3A: it is not necessary to include any additional experimentation in this figure; however, while the main text points out that the sasA/B double mutant is defective for ppGpp after amino acid downshift, the double mutant is not shown in Fig 3A for comparison, instead it is shown in Fig S5. The data can all be found in the current manuscript, and it therefore does not require any experimental changes. That being said, the location of the data in S5 is a bit odd.

The Δ*sasA*Δ*sasB* double mutant was not included in Fig. 3A because those experiments were run at different times. We thought it would be a misrepresentation to include those plots all in the same panel.

Fig 8A: error measurements are included for the luciferase reporter but not for the growth data. It is a minor comment, but is there a specific reason for that?

This was an oversight and has been corrected. See changes to Fig. 8A and S13.
