# Supplementary material for: Analysis of (p)ppGpp metabolism and signaling using a dynamic luminescent reporter
Source: PLoS Genet. 2025 Aug 22;21(8):e1011691. doi: 10.1371/journal.pgen.1011691 (PMC12373219; doi:10.1371/journal.pgen.1011691)
Supplement: Attachment — Submitted filename: Rebuttal rev 2.docx [file pgen.1011691.s020.docx]

**Reviewers' comments:**

Reviewer's Responses to Questions

Reviewer #1: The authors have addressed all my concerns. I recommend the publication of the manuscript.

Reviewer #2: The authors have addressed most of my prior review concerns and questions, however there are some minor aspects that remain:

R2 Point #3: "Second, while (p)ppGpp has a clear inhibitory effect on global protein synthesis (54), this effect would tend to dampen the response of a protein reporter such as luciferase thereby minimizing observed (p)ppGpp-dependent increase in RsFluc activity." The original review point was not about the observed luc signal spike, which this reviewer did not doubt is due to a rise in ppGpp, but instead whether the subsequent DROP in luciferase signal could be due to ppGpp-dependent inhibition of global protein synthesis. Meaning, ppGpp levels might remain high for longer than is observed by the luc reporter. Notably, the ppGpp-0 control cannot rule this out. In the revision, to address this experimentally, the authors have included quantitation of the reporter mRNA by FISH, shown in Fig. S4. However, they need to address the following issues:

(a) Fig. S4A - the x-axis is missing the timepoint values. In particular, without knowing the time difference between TP3 and TP4, it is not possible to evaluate whether the mRNA FISH data make sense with the reporter luminescence data. These results also bear more discussion, because they suggest that ppGpp levels are already low at TP4, which is when luc reporter signal is highest, so there appears to be a time-lag that should be noted. Also, ppGpp levels dropping should not affect the stability of existing full-length transcripts, so depending on the time difference between TP3 and TP4, the observed FISH result may be surprising.

Thank you for your comment! We have revised the figure to include time values and gridlines, as well as added additional information about the sampling times to the figure legend. We have also added comment on the time scale to further clarify. See lines 134-137.

(b) Description of new FISH experiment in the Methods section does not describe how the fluorescence was quantitated. The level of statistical also is not described (how many cells; how many probes).

Microscopy image quantification methods are included in the microscopy section of the methods. See lines 539-542. Figure legend has been updated to include the number of cells sampled at each time point. See Table S3 for oligo probe info and number.
